# RETRACTION: The Efficacy of Electroacupuncture in Among Early Diabetic Patients with Lower Limb Arteriosclerotic Wounds

**DOI:** 10.1111/iwj.70478

**Published:** 2025-04-02

## Abstract

**RETRACTION:** SangP. , ZhaoJ. , and YangH. , “The Efficacy of Electroacupuncture in Among Early Diabetic Patients with Lower Limb Arteriosclerotic Wounds,” International Wound Journal 21, no. 4 (2024): e14526, 10.1111/iwj.14526.38093499 PMC10961040

The above article, published online on 13 December 2023, in Wiley Online Library (http://onlinelibrary.wiley.com/), has been retracted by agreement between the journal Editor in Chief, Professor Keith Harding; and John Wiley & Sons, Ltd. Following an investigation by the publisher, all parties have concluded that this article was accepted solely on the basis of a compromised peer review process. The editors have therefore decided to retract the article. The authors did not respond to our notice regarding the retraction.

**RETRACTION:** P. Sang , J. Zhao , and H. Yang , “The Efficacy of Electroacupuncture in Among Early Diabetic Patients with Lower Limb Arteriosclerotic Wounds,” International Wound Journal 21, no. 4 (2024): e14526, 10.1111/iwj.14526.38093499 PMC10961040

The above article, published online on 13 December 2023, in Wiley Online Library (http://onlinelibrary.wiley.com/), has been retracted by agreement between the journal Editor in Chief, Professor Keith Harding; and John Wiley & Sons, Ltd. Following an investigation by the publisher, all parties have concluded that this article was accepted solely on the basis of a compromised peer review process. The editors have therefore decided to retract the article. The authors did not respond to our notice regarding the retraction.

